# Supplementary figures and images for: NPC Intracellular Cholesterol Transporter 1 Regulates Ovarian Maturation and Molting in Female Macrobrachium nipponense
Source: Int J Mol Sci. 2024 May 31;25(11):6049. doi: 10.3390/ijms25116049 (PMC11172811; doi:10.3390/ijms25116049)

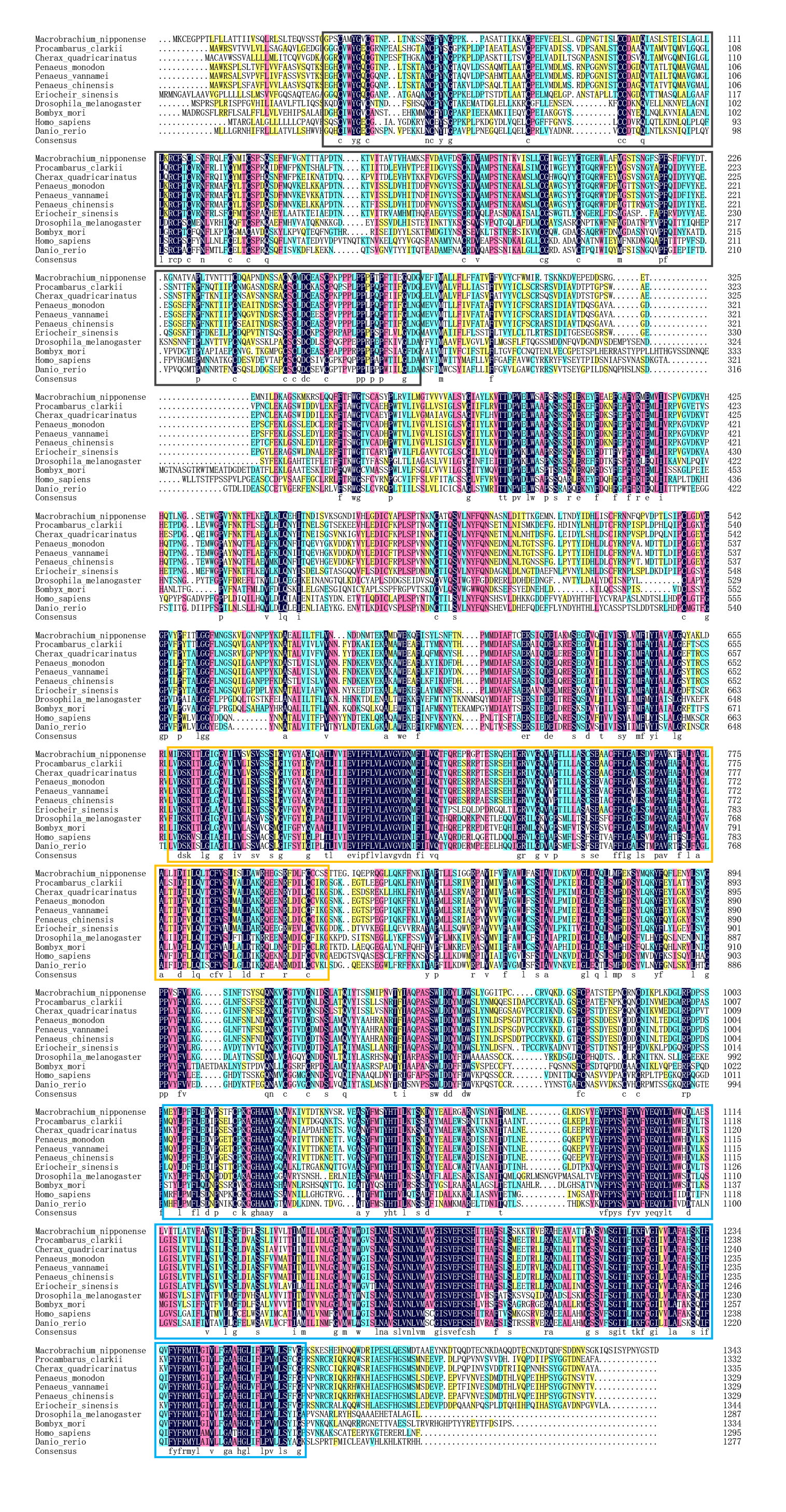

Supplement: Supplementary file 1 [file ijms-25-06049-s001.zip › Figure S2.tif]
